# Nomograms forecasting long‐term overall and cancer‐specific survival of patients with oral squamous cell carcinoma

**DOI:** 10.1002/cam4.1216

**Published:** 2018-03-07

**Authors:** Fengze Wang, Hui Zhang, Jiao Wen, Jun Zhou, Yuan Liu, Bingkun Cheng, Xun Chen, Jianhua Wei

**Affiliations:** ^1^ Department of stomatology The 316th Hospital of Chinese People's Liberation Army No. A2 Niangniangfu, Xiangshan Road Beijing Haidian District China; ^2^ State Key Laboratory of Military Stomatology & National Clinical Research Center for Oral Diseases & Shaanxi Clinical Research Center for Oral Diseases Department of Oral and Maxillofacial Surgery School of Stomatology The Fourth Military Medical University Xi'an China; ^3^ State Key Laboratory of Military Stomatology & National Clinical Research Center for Oral Diseases & Shaanxi Engineering Research Center for Dental Materials and Advanced Manufacture Department of Anesthesiology School of Stomatology The Fourth Military Medical University Xi'an China; ^4^ State Key Laboratory of Military Stomatology & National Clinical Research Center for Oral Diseases & Shaanxi International Joint Research Center for Oral Diseases Department of Oral Histology and Pathology The Fourth Military Medical University Xi'an China; ^5^ Department of oral and maxillofacial surgery The Second Hospital of Hebei Medical University Shijiazhuang China

**Keywords:** Cancer‐specific survival, nomogram, oral squamous cell carcinoma, overall survival, SEER

## Abstract

Our aim was to establish a “nomogram” model to forecast the overall survival (OS) and cancer‐specific survival (CSS) of oral squamous cell carcinoma (OSCC) patients. The clinicopathological data for the 10,533 OSCC patients were collected from the Surveillance, Epidemiology and End Results (SEER) database. We used a credible random split‐sample method to divide 10,533 patients into two cohorts: 7046 patients in the modeling cohort and 3487 patients in the external validation cohort (split‐ratio = 2:1). The median follow‐up period was 32 months (1–119 months). We developed nomograms to predict 5‐ and 8‐year OS and CSS of OSCC patients with a Cox proportional hazards model. The precision of the nomograms was assessed by the concordance index (C‐index) and calibration curves through internal and external validation. The C‐indexes of internal validation regarding 5‐ and 8‐year OS and CSS were 0.762 and 0.783, respectively. In addition, the external validation's C‐indexes were 0.772 and 0.800. Based on a large‐sample analysis targeting the SEER database, we established two nomograms to predict long‐term OS and CSS for OSCC patients successfully, which can assist surgeons in developing a more effective therapeutic regimen and conducting personalized prognostic evaluations.

## Introduction

Oral squamous cell carcinoma (OSCC) is ranked as the eighth most common malignant cancer worldwide according to the WHO global oral health programme 1. The incidence of OPSCC ranges from 1/10,0000 to 10/10,0000 in the world, which will likely increase gradually in the coming years 2, 3. It is worth noting that the mortality of OSCC patients in developing countries was higher than their counterparts in developed countries 2. Moreover, in the GLOBOCAN 2008 report, 128000 deaths were caused by OSCC worldwide in 2008 4. Therefore, it is imperative to develop an accurate model to evaluate OSCC patient prognosis.

Currently, the National Comprehensive Cancer Network (NCCN) clinical practice guidelines recommended assessing a prognosis via the American Joint Committee on Cancer (AJCC) Staging Manual (7th edition) 5, 6. However, the outcome of OSCC patients is influenced by many other factors such as age, sex, race, tumor site, radiation, and surgery 2, 7, 8, 9. Hence, consideration of other relevant clinicopathological factors could provide a more credible prediction result than the AJCC staging manual. Therefore, we sought to construct a “nomogram” to identify other elements including age, sex, tumor site, pathological grade, surgery or not, radiation or not and TNM classifications. The nomograms were established with a popular random split‐sample method 10, 11, 12. Moreover, researchers obtained a favorable model via a split‐ratio of 1:1 and 1:2 13. Nomograms were validated internally and externally through the concordance index (C‐index) and calibration plot 12.

A nomogram's construction was based on the independent prognostic factors via Kaplan‐Meier and multivariate Cox proportional hazards model. Currently, a nomogram has been widely used to assist surgeons in developing treatment plans and evaluating the prognosis of cancers such as hepatocellular carcinoma 14, gastric cancer 15, nasopharyngeal cancer 16 and breast cancer 17. Most importantly, the early detection of prostate cancers via nomogram had been written into the NCCN clinical guidelines 18. Hence, we sought to establish two nomograms to assess the 5‐ and 8‐year OS and CSS based on the SEER database to provide a reference to surgeons.

## Materials and Methods

### Patients' general information

We collected the clinicopathological data of all 10,533 OSCC patients from the years 2004 to 2012 from the SEER program of the National Cancer Institute 19. The detailed information included age, sex, race, ethnic origin, tumor site, grade, surgery, radiation, T stage, N stage and M stage (Table 1). The minimum age was 15 years old. The racial composition consisted of white, black and others (American Indian/AK Native, Asian/Pacific Islander). Tumor sites comprised hard palate, cheek, mouth floor and tongue (excluded tongue base).

**Table 1:** Patients' clinicopathological data

Variables	Modeling group (*n* = 7046)		Validation group (*n* = 3487)	
	*n*	%	*n*	%
Age				
15–35	285	4.0	161	4.6
36–45	615	8.7	301	8.6
46–55	1606	22.8	816	23.4
56–65	2001	28.4	966	27.7
66–75	1402	19.9	671	19.2
76–85	842	12.0	440	12.6
85+	292	4.2	132	3.8
Sex				
Male	4224	59.9	2148	61.6
Female	2825	40.1	1339	38.4
Site				
HP	445	6.3	212	6.1
Cheek	665	9.4	353	10.1
MF	1622	23.0	774	22.2
Tongue	4314	61.2	2148	61.6
Race				
White	5776	82.0	2902	83.2
Black	595	8.4	252	7.2
Others	675	9.6	333	9.5
Origin				
NSHL	6444	91.5	3193	91.6
SHL	602	8.5	294	8.4
Grade				
I	1683	23.9	813	23.3
II	4050	57.5	2020	57.9
III	1278	18.1	628	18.0
IV	35	0.5	26	0.7
Surgery				
Performed	6079	86.3	3034	87.0
None	967	13.7	453	13.0
Radiation				
Yes	3194	45.3	1565	44.9
No	3852	54.7	1922	55.1
T stage				
T1	3273	46.5	1592	45.7
T2	1981	28.1	1059	30.4
T3	723	10.3	339	9.7
T4	1069	15.2	497	14.2
N stage				
N0	4626	65.7	2335	67.0
N1	1001	14.2	483	13.9
N2	1352	19.2	637	18.2
N3	67	1.0	32	0.9
M stage				
M0	6941	98.5	3442	98.7
M1	105	1.5	45	1.3

HP, Hard Palate; MF, Mouth Floor. Others, American Indian/AK Native, Asian/Pacific Islander; NSHL, Nonspanish‐Hispanic‐Latino; Grade I, Well differentiated; II, Moderately differentiated; III, Poorly differentiated; IV, Undifferentiated.

### Survival analysis

We also obtained the survival data by searching with the SAS name “srv_time_mon”, “STAT_REC”, “VSRTSADX”. The SAS names “STAT_REC”, “VSRTSADX” represented the overall survival (OS) and cancer‐specific survival (CSS) of patients, respectively. We excluded the patients whose information was collected from autopsy and death certificates. We conducted OS and CSS analyses using the Kaplan‐Meier and Cox Proportional hazards models, which is consistent with a research study published in JAMA Oncol 20. All statistical analyses were performed applying a two‐sided *P* value and *P* < 0.05 was considered statistically significant.

### Nomogram development

We acquired the independent prognostic factors with regard to OS and CSS of OSCC patients by virtue of SPSS 21.0 software for Windows. We conducted the nomograms via the “cmprsk package” of R software version 3.2.4.

### Nomogram validation

The nomogram's accuracy was required to be validated by 1000 times bootstrapping and 10‐fold cross‐validation measures internally and externally. The fitting degree was evaluated by concordance indexes (C‐index) and calibration plots 12. The C‐index and calibration were obtained by the “rcorrcens” and “calibrate” commands in R software. In addition, the calibration plot consisted of two lines: one was a 45‐degree reference line, and the other line represented the actual line. The interval between the two lines reflected the accuracy of the nomograms.

## Results

### Patient clinicopathological data

After strict filtering based on the SEER database, 7046 and 3487 OSCC patients were included in the modeling and validation cohorts, respectively, via the popular random split‐sample method (the split ratio was 2:1). The patients' ages ranged from 15 to 96 years (median, 55) in the modeling cohort. Of these, 7046 OSCC patients, 4224(59.9%) were male. A total of 5776 (82.0%) patients were white, and 6444 (91.5%) patients were non‐Spanish‐Hispanic‐Latino. With regard to tumor sites, 4314(61.2%) tumors were located on the tongue (excluded tongue base) and 1622 (23.0%) were primarily found on the mouth floor. In addition, 5733 (81.4%) were well and moderately defined. Of these cases, 6079 (86.3%) received surgery and 3194 (45.3%) underwent radiotherapy. The proportions of T1–T2 and T3–T4 were 74.6% (5254/7046) and 25.5% (1792/7046), respectively. The N0 and M0 tumors accounted for 65.7% and 98.5% of the total specimens, respectively. The detailed information for the validation cohort is shown in Table 1.

### Survival analysis and nomogram construction

In terms of the SAS variable “sur_time_mon” in the SEER database, we found that the median follow‐up periods of the modeling and validation cohorts were 31 months (1–119 months) and 33 months (1–119 months). According to the SAS variables “STAT_REC” and “VSRTSADX” in SEER database, we acquired credible data on the overall survival (OS) and cancer‐specific death (CSD) for 10533 OSCC patients. In total, 3064 (43.5%) patients in the modeling cohort were deceased at the last follow‐up date. Among those patients, 2196 (31.2%) patients died due to OSCC. Additionally, 868 (12.3%) patients died due to other causes rather than OSCC.

**Figure 1:**
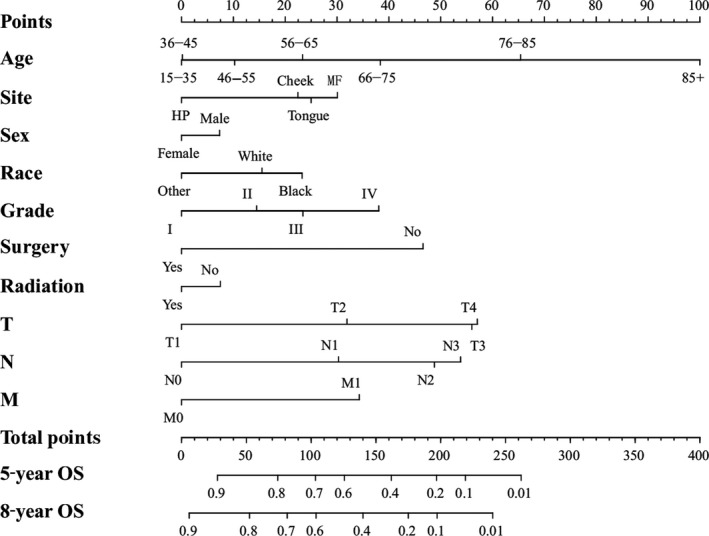
Nomogram predicting 5‐year and 8‐year OS. HP, Hard Palate; MF, Mouth Floor; Others, American Indian/Alaska Native/Asian or Pacific Islander; Grade I, Well differentiated; II, Moderately differentiated; III, Poorly differentiated; IV, undifferentiated.

**Figure 2:**
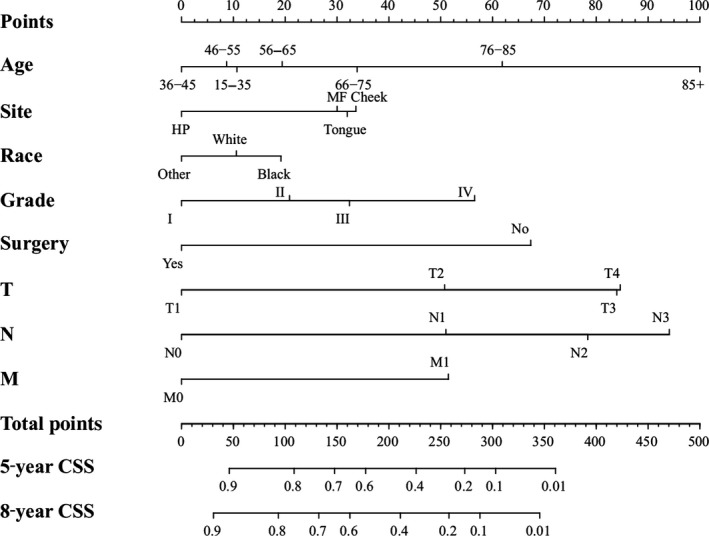
Nomogram predicting 5‐year and 8‐year CSS. HP, Hard Palate; MF, Mouth Floor; Others, American Indian/Alaska Native/Asian or Pacific Islander; Grade I, Well differentiated; II, Moderately differentiated; III, Poorly differentiated; IV, undifferentiated. ***Nomogram validation:*** The nomograms were validated by bootstrap resampling and tenfold cross‐validation methods. The Harrell concordance index (C‐index) and calibration curves were used to evaluate the accuracy of the nomograms internally and externally. The predicted OS and CSS conformed to the actual OS and CSS if the value of C‐index was greater than 0.7. Our results of internal validation indicated that the C‐index values of OS and CSS were 0.762 and 0.783, respectively. External validation showed that the C‐index value of OS and CSS increased slightly to 0.773 and 0.800. Additionally, the internal and external calibration curves approached the 45‐degree ideal straight line (Figs. 3 and 4).

We conducted the univariate and multivariate analysis targeting overall survival (OS) and cancer‐specific survival (CSS) via SPSS 21.0 software for Windows (Table 2 and 3). The results of univariate and multivariate survival analyses showed that age, sex, tumor sites, race, pathological grade, surgery, radiation and TNM staging were independent prognostic factors, which showed statistical significance (*P *< 0.05). Then, we established a nomogram to take all these elements into account, as shown in Figure 1. Meanwhile, we carried out an analysis focusing on cancer‐specific survival using SPSS software. The results of univariate and multivariate survival analyses indicated that age, site, race, pathological grade, surgery and TNM classifications were the independent risk elements influencing the prognosis. Furthermore, we constructed another nomogram predicting the 5‐ and 8‐year CSS (Fig. 2).

**Table 2:** Univariate and multivariate analyses of OS in nomogram cohort

		Multivariate analysis	
Variables	Univariate analysis *P* value	HR (95% CI)	*P* value
Age	<0.001		<0.001
15–35		0.202 (0.154–0.266)	<0.001
36–45		203 (0.166–0.249)	<0.001
46–55		0.239 (0.203–0.282)	<0.001
56–65		0.295 (0.252–0.345)	<0.001
66–75		0.376 (0.321–0.440)	<0.001
76–85		0.579 (0.493–0.679)	<0.001
85+		Reference	
Sex	<0.001		0.002
Male		Reference	
Female		0.888 (0.823–0.959)	0.002
Site	<0.001		<0.001
HP		0.672 (0.567–0.797)	<0.001
Cheek		0.960 (0.848–1.088)	0.526
MF		1.085 (0.996–1.181)	0.061
Tongue		Reference	
Race	<0.001		<0.001
White		Reference	
Black		1.132 (1.006–1.275)	0.040
Others		0.781 (0.681–0.896)	<0.001
Origin	0.281		
NSHL			
SHL			
Grade	<0.001		<0.001
I		0.546 (0.344–0.867)	0.010
II		0.689 (0.437–1.087)	0.109
III		0.795 (0.502–1.259)	0.328
IV		Reference	
Surgery	<0.001		<0.001
Performed		Reference	
None		2.090 (1.905–2.293)	<0.001
Radiation	<0.001		<0.001
Yes		Reference	
No		1.119 (1.026–1.221)	0.011
T stage	<0.001		<0.001
T1		0.403 (0.358–0.452)	<0.001
T2		0.671 (0.604–0.744)	<0.001
T3		0.983 (0.873–1.106)	0.772
T4		Reference	
N stage	<0.001		<0.001
N0		0.426 (0.319–0.570)	<0.001
N1		0.691 (0.515–0.925)	0.013
N2		0.925 (0.694–1.233)	0.602
N3		Reference	
M stage	<0.001		<0.001
M0		0.586 (0.472–0.728)	<0.001
M1		Reference	

HP, Hard palate; MF, mouth floor. Others: American Indian/AK Native, Asian/Pacific Islander. NSHL: Nonspanish‐Hispanic‐Latino. Grade I: Well differentiated. II: Moderately differentiated. III: Poorly differentiated. IV: Undifferentiated.

**Table 3:** Univariate and multivariate analyses of CSS in nomogram cohort

		Multivariate analysis	
Variables	Univariate analysis *P* value	HR (95% CI)	*P* value
Age			
15–35		0.361 (0.268–0.485)	<0.001
36–45		0.322 (0.255–0.407)	<0.001
46–55		0.363 (0.299–0.441)	<0.001
56–65		0.407 (0.337–0.492)	<0.001
66–75		0.486 (0.401–0.590)	<0.001
76–85		0.664 (0.543–0.812)	<0.001
85+		Reference	
Sex	0.136		
Male			
Female			
Site	<0.001		0.004
HP		0.685 (0.559–0.840)	<0.001
Cheek		1.021 (0.884–1.179)	0.777
MF		0.977 (0.883–1.082)	0.655
Tongue		Reference	
Race	<0.001		0.037
White		Reference	
Black		1.112 (0.971–1.274)	0.125
Others		0.862 (0.739–1.006)	0.060
Origin	0.108		
NSHL			
SHL			
Grade	<0.001		<0.001
I		0.535 (0.317–0.902)	0.019
II		0.699 (0.418–1.169)	0.172
III		0.799 (0.476–1.342)	0.397
IV		Reference	<0.001
Surgery			<0.001
Performed		Reference	
None		2.260 (2.032–2.514)	<0.001
Radiation	<0.001		0.252
Yes		Reference	
No		1.062 (0.958–1.178)	0.252
T stage	<0.001		<0.001
T1		0.364(0.318–0.417)	<0.001
T2		0.669 (0.594–0.753)	<0.001
T3		0.992 (0.869–1.133)	0.906
T4		Reference	
N stage	<0.001		<0.001
N0		0.328 (0.242–0.445)	<0.001
N1		0.603 (0.444–0.821)	0.001
N2		0.834 (0.618–1.125)	0.235
N3		Reference	
M stage	<0.001		<0.001
M0		0.549 (0.434–0.693)	<0.001
M1		Reference	

HP, Hard palate; MF, Mouth Floor. Others: American Indian/AK Native, Asian/Pacific Islander. NSHL, Nonspanish–Hispanic–Latino. Grade I: Well differentiated. II: Moderately differentiated. III: Poorly differentiated. IV: Undifferentiated.

## Discussion

OSCC was ranked as the eighth most prevalent cancer in the world 21. Due to the higher incidence and mortality of OSCC according to the statistics of the World Health Organization, oral cancers became the burden of public health 2. Most importantly, the WHO Global Oral Health Programme constructed a global surveillance system to evaluate the risk factors of OSCC 2. Generally, surgery and radiation were the main measures to address OSCC 5, 22. However, in this large‐sample retrospective research, we found that 1420 (13.5%) and 5774 (54.8%) cases did not receive surgery and radiation, respectively. Meanwhile, early detection of OSCC was still a serious problem 23. Current studies indicated that more OSCC patients were diagnosed with an advanced stage, influencing the survival of OSCC patients seriously 24. Thus, it is imperative to establish an accurate prediction model to guide surgeons to conduct OSCC's early detection and prognostic evaluation individually. Additionally, the 8th AJCC staging system indicated that they would assess the prognosis by taking consideration the nomogram in the future version 25. Hence, establishing a credible nomogram prediction model remained a top priority.

We calculated the estimated overall survival (OS) and cancer‐specific survival (CSS) via Kaplan‐Meier method, which was consistent with the research published in JAMA Oncol 20. In the survival analysis of OS, we found that the OS of females was higher than males (Fig. 1 and Table 2). Meanwhile, the female's CSS was superior to male, although it did not show statistical significance (Table 3). The results above were consistent with retrospective research in the United Kingdom 26. We hypothesized that males were susceptible to indulge in smoking and drinking alcohol, which was closely linked to OSCC 27. In terms of age, we found that the OS and CSS descended beyond the year of 55. Those aged “15–35” and “36–45” had an improved OS and CSS, respectively. Current results have shown that the majority of OSCC patients were diagnosed after the age of 50 9. Our results indicated that the OS and CSS of black OSCC patients were lower than that of other races, which was consistent with the research 28. One research study hypothesized that melanin might contribute to the development of OSCC 29. The OSCC patients had an improved OS and CSS after surgery and radiation therapy (Figs. 1 and 2; Tables 2 and 3)

**Figure 3:**
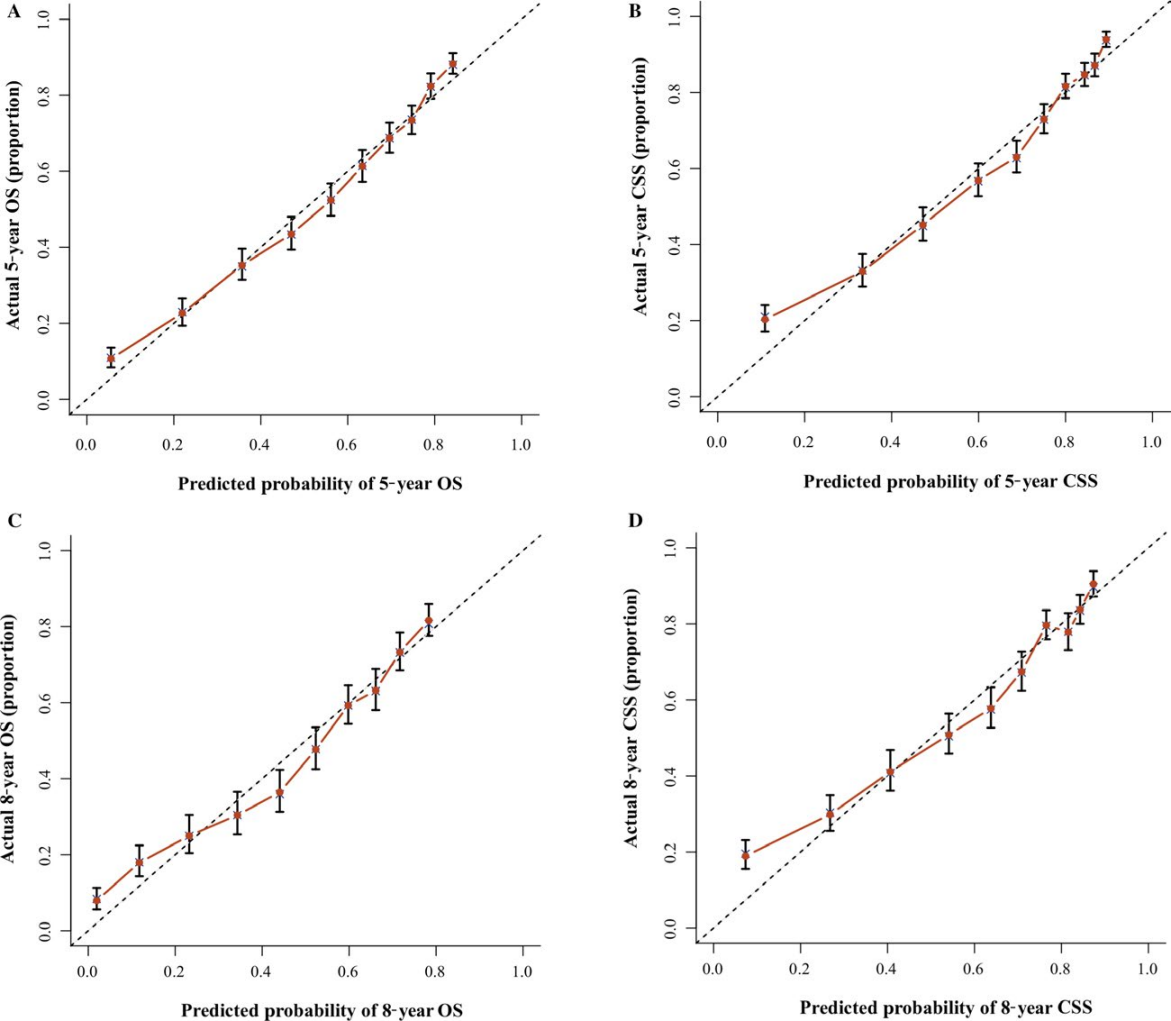
Internal calibration nomogram for 5‐year and 8‐year OS (A, C) and 5‐year and 8‐year CSS (B, D). The 45‐degree line represents an ideal match between the actual survival (*Y*‐axis) and nomogram‐predicted survival (*X*‐axis). The perpendicular line means 95% confidence intervals.

**Figure 4:**
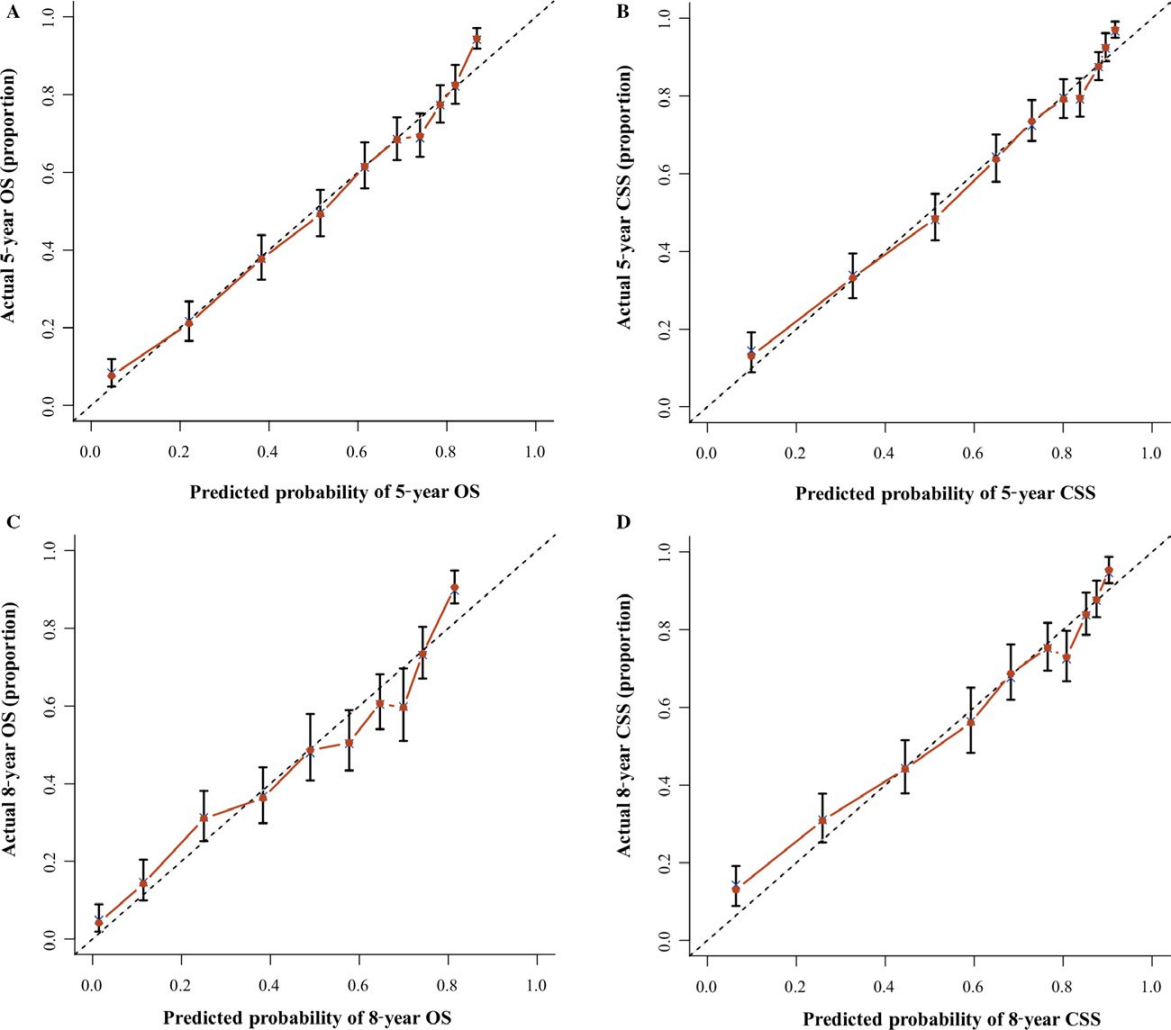
External calibration nomogram for 5‐year and 8‐year OS (A, C) and 5‐year and 8‐year CSS (B, D). The 45‐degree line represents an ideal match between the actual survival (*Y*‐axis) and nomogram‐predicted survival(*X*‐axis). The perpendicular line means 95% confidence intervals.

We validated the accuracy of nomograms internally (modeling cohort) and externally (validation cohort) by virtue of C‐index and calibration curves. We split the total 10533 specimens with a random split‐sample method and the split‐ratio was 2:1, which was in accordance with the research 10. The C‐indexes of internal validation regarding 5‐ and 8‐year OS and CSS were 0.762 and 0.783, respectively. In addition, the external validation's C‐indexes were 0.772 and 0.800. The values of the C‐index were all greater than 0.7 and there was excellent coherence between the calibration curves and the 45‐degree ideal lines (Figs. 3 and 4).

The process of creating nomograms to forecast the 5‐ and 8‐year OS and CSS was simple and feasible. First, we drew vertical lines from the clinicopathological factors to the axes of the points. After acquiring the total points, we plotted the vertical lines from the total points to the axes of 5‐ and 8‐year OS and CSS to obtain the predicted values. Most importantly, using the nomogram to predict a prognosis was more accurate than using the AJCC staging manual. For example, two types of T3N0M0 OSCC patients: type 1, a 55‐year‐old black male patient with grade IV disease who received surgery and radiation; and type 2, a 60‐year‐old white female patient with grade II disease underwent surgery only. The prognoses of these two types of patients were identical if we used the AJCC staging manual 6. However, the results were different via the nomogram. The 5‐year predicted OS for the type 1 and type 2 patients were 57% and 68%, respectively. Moreover, the CSS of the type 1 and 2 patients was 64% and 81%, respectively. Hence, we constructed two accurate nomogram models to predict the prognoses of the OSCC patients.

Our research had strengths and certain limitations. We completed a large‐sample retrospective study based on the SEER database and succeeded in establishing more accurate nomogram models. A lot of researches had shown that other relevant clinicopathological factors were influenced the survival of patients with oral cancers such as HPV 30, nodal involvement 31, thickness of the tumor 32, P53 33, EGFR 34, cigarette and alcohol consumption 35, and chemotherapy 5. However, the SEER database didn't include these elements above. For the same reason, we could not evaluate disease‐free survival and loco‐regional control. Hence, our nomogram could not assess these factors. We will conduct a prospective study to detect these indicators to remedy these limitations.

In conclusion, we conducted the survival analysis conscientiously and succeeded in establishing two accurate nomograms, which can provide surgeons with a reference to tailor clinical therapeutic regimens and provide a personalized prognosis.

## Conflicts of Interests

None declared.
